# Application of Benchtop-magnetic resonance imaging in a nude mouse tumor model

**DOI:** 10.1186/1756-9966-30-69

**Published:** 2011-07-21

**Authors:** Henrike Caysa, Hendrik Metz, Karsten Mäder, Thomas Mueller

**Affiliations:** 1 Martin-Luther-University Halle-Wittenberg, Department of Pharmaceutics and Biopharmaceutics, Wolfgang-Langenbeck-Str. 4, 06114 Halle/Saale, Germany; 2 Martin-Luther-University Halle-Wittenberg, Department of Internal Medicine IV, Oncology/Hematology, Ernst-Grube-Str. 40, 06120 Halle/Saale, Germany

## Abstract

**Background:**

MRI plays a key role in the preclinical development of new drugs, diagnostics and their delivery systems. However, very high installation and running costs of existing superconducting MRI machines limit the spread of MRI. The new method of Benchtop-MRI (BT-MRI) has the potential to overcome this limitation due to much lower installation and almost no running costs. However, due to the low field strength and decreased magnet homogeneity it is questionable, whether BT-MRI can achieve sufficient image quality to provide useful information for preclinical in vivo studies. It was the aim of the current study to explore the potential of BT-MRI on tumor models in mice.

**Methods:**

We used a prototype of an in vivo BT-MRI apparatus to visualise organs and tumors and to analyse tumor progression in nude mouse xenograft models of human testicular germ cell tumor and colon carcinoma.

**Results:**

Subcutaneous xenografts were easily identified as relative hypointense areas in transaxial slices of NMR images. Monitoring of tumor progression evaluated by pixel extension analyses based on NMR images correlated with increasing tumor volume calculated by calliper measurement. Gd-BOPTA contrast agent injection resulted in a better differentiation between parts of the urinary tissues and organs due to fast elimination of the agent via kidneys. In addition, interior structuring of tumors could be observed. A strong contrast enhancement within a tumor was associated with a central necrotic/fibrotic area.

**Conclusions:**

BT-MRI provides satisfactory image quality to visualize organs and tumors and to monitor tumor progression and structure in mouse models.

## Background

MRI plays a key role in the preclinical development of new drugs, diagnostics and their delivery systems. However, very high installation and running cost of existing superconducting MRI machines limit the spread of the method. The new method of Benchtop-MRI (BT-MRI) has the potential to overcome this limitation due to much lower installation and almost no running costs. The lower quality of the NMR images is expected due to the low field strength and decreased magnet homogeneity. However, very recently we could show that BT-MRI is able to characterize floating mono- or bilayer tablets, osmotic controlled push-pull tablets [1-4] or scaffolds for tissue engineering in vitro [5]. A broad, important and increasing range of MRI applications are linked with preclinical studies on small rodents such as mice or rats [6-8]. Thereby, first developments and testing of more compact MRI systems have been reported [9,10]. In the present study we have tested a prototype of a new in vivo BT-MRI apparatus.

Clearly, BT-MRI could overcome one of the current main limitations of preclinical MRI, the high costs. However, the question arises, whether BT-MRI can achieve sufficient image quality to provide useful information for preclinical in vivo studies. In a recent paper we have demonstrated that BT-MRI can be used to characterize in situ forming implants in mice [11]. A major application field of preclinical MRI is linked to cancer research. It was therefore the aim of the current study to explore the potential of BT-MRI on tumor models in mice. Nude mouse xenograft models of different human tumors were used to test the suitability of the new BT-MRI system for visualisation of organs and tumors and for quantification of tumor progression.

## Methods

### NMR system and its characteristics

**Figure 1:**
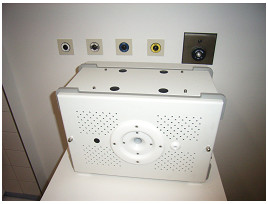
**Prototype of the Benchtop-MRI system "MARAN DRX2" (Oxford Instruments)**.

A 21 MHz NMR benchtop prototype system "MARAN DRX2" (Oxford Instruments) capable of imaging with a horizontal bore of 23 mm diameter was used (Figure 1). The instrument is equipped with a temperature control unit and capable of T_1 _and T_2 _relaxation measurements, the determination of diffusion coefficients and imaging.

### NMR imaging parameter

The temperature was set to 37°C. Always 4 slices were simultaneously measured with: slice distance: 3.5 mm, slice width: 3 mm, spin echo time TE: 9.8 ms, repetition time TR: 172 ms, averages: 32 or 16 (for time critical kinetics), total time: 715 s or 357 s, respectively, FOV: 40*40 mm. The pulse sequence was T2SE.

The MRI acquisition parameters were optimized under some hardware restrictions. TE is limited by the bandwidth of 10 KHz to 9.8 ms. An increase of the bandwidth allows shorter TE, however it leads also to stronger image distortions. A TR value of 150 ms gives an optimal contrast for marbled meat and also for mice. For 4 slices TR is limited to 171.4 ms. Therefore 172 ms was used for TR as a good compromise between best contrast and simultaneous acquisition of 4 slices. The resulting images are therefore T1-weighted and range from hyperintense signals for fatty tissues to hypointense signals for water. The higher number of averages was chosen to improve the signal-to-noise ratio. For kinetics of contrast agent distribution a rapid image acquisition may be essential. Therefore measurements with lesser averages were also performed, even though the image quality is reduced.

### Cell culture, xenograft tumor model, measurements and analyses

Human colon carcinoma cell lines DLD-1, HCT8 and HT29 and human testicular germ cell tumor cell line 1411HP were maintained as monolayer cultures in RPMI-1640 with 10% FCS and streptomycin/penicillin. Cultures were grown at 37°C in a humidified atmosphere of 5% CO_2_/95% air.

Eight week old male athymic-nude Foxn1 nu/nu mice (Harlan Winkelmann, Germany) were injected s.c. with 3 × 10^6 ^tumor cells in both flanks. NMR Imaging of mice was performed once a week. For comparison, the size of the xenograft tumors was also measured by means of a calliper. For imaging with a positive MRI contrast agent mice received 150 μl of gadobenate dimeglumine (Gd-BOPTA; 0.03 mmol/kg in 0.9% NaCl) via tail vein injection. For investigation of contrast agent associated effects with special focus on xenograft tumors the dose of Gd-BOPTA was increased according to dosage applied in men (0.1 mmol/kg). Animals were anaesthetised via i.p. application of ketamine/xylazine mixture prior to imaging. Body weight was assessed twice weekly. For histological examination tumors were explanted, fixed in 4% formalin and embedded in paraffin. Hematoxylin/Eosin staining of slices was performed according to standard protocols. All animal protocols were approved by the laboratory animal care and use committee of Sachsen-Anhalt, Germany.

Quantification of xenograft tumor growth was performed by

1.) volume calculation based on calliper measurements using the formula **a^2 ^× b × π/6 **with **a **being the short and **b **the long dimension and

2.) measurement of pixel extensions of tumor sections based on NMR images (128 × 128 JPG) using the measure tool of GNU Image Manipulation Program (GIMP 2.6.8) and calculating the area using formula **A = a/2 × b/2 × π**.

## Results

### Imaging of organs and tumors; gadobenate dimeglumine (Gd-BOPTA) induced MRI contrast

**Figure 2:**
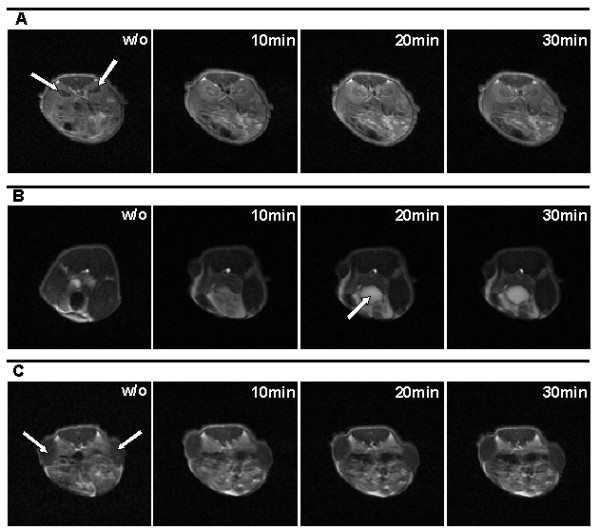
**Transaxial NMR images of mice (face-down position) bearing two s.c. xenografts; left: 1411HP germ cell tumor, right: DLD-1 colon carcinoma**. Images were taken without Gd-BOPTA and 10 min, 20 min and 30 min after i.v. application of Gd-BOPTA. **(A): **The illustration of renal pelvis was clearly enhanced directly after contrast agent injection in light grey compared to a black central area without Gd-BOPTA. The fast nephritic elimination caused a signal decrease (darker grey) already after 30 min. White arrows point at kidneys. **(B): **High contrast enhancement in the urinary bladder (white arrow) was identifiable as hypertense area compared to a hypotense one without contrast agent. **(C): **Subcutaneous xenograft tumors are visible as relative hypointense area (white arrows).

A nude mouse xenograft model of different human tumors was used to determine the image sensitivity and quality of the BT-MRI system. Gd-BOPTA as one of the clinically used low molecular weight gadolinium chelates was selected for contrast agent enhanced MRI. A good differentiation between cortex of kidney and renal pelvis could be observed depending on circulation time of the contrast agent (Figure 2A). Furthermore, the fast renal elimination of Gd-BOPTA was visualised. The urinary bladder was visible as a bright, hypertense sphere unlike the NMR image without contrast agent (Figure 2B). Subcutaneous xenograft tumors were easily identified as relative hypointense area at each body site (Figure 2C).

**Figure 3:**
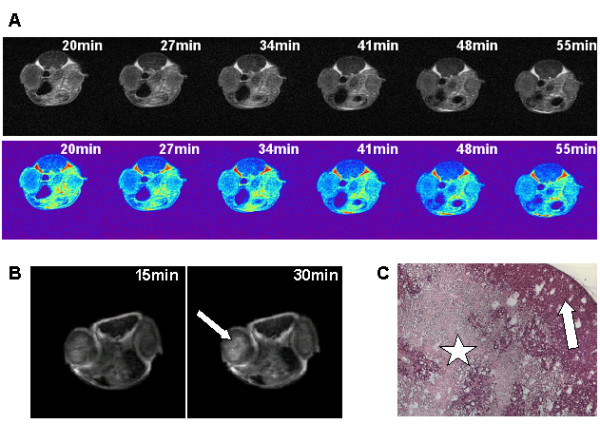
**Analysis of contrast agent induced interior structuring of tumours**. **(A):** Transaxial NMR images of a mouse (face-down position) bearing two s.c. xenografts; left: HT29 colon carcinoma, right HCT8 colon carcinoma. Images were taken to the indicated time points after i.v. application of higher dosed Gd-BOPTA (0.1 mmol/kg). A time dependent alteration of contrast enhancement with initial enhancement of the tumor rim followed by a centripetal progression of the signal is observed in the HT29 tumor. The HCT8 tumor was too small for detailed analyses although a time dependent alteration of the signal could also be observed. (upper panel - grayscale, lower panel - pseudocolor) **(B): **Transaxial NMR images of a mouse (face-down position) bearing two s.c. HT29 xenografts 15 min and 30 min after i.v. application of Gd-BOPTA. One tumor showed strong contrast enhancement and an interior structuring could be observed (white arrow). **(C): **HE staining of the well structured left HT29 xenograft shown in (A). Depicted is a section at the side of the tumor to represent the whole structure composed of a large central necrotic/fibrotic area (white star) surrounded by peripherally arranged vital tumor cells (white arrow).

To study the contrast agent associated effects with special focus on xenograft tumors we used a higher dose of Gd-BOPTA according to dosage applied in men. As shown in Figure 3A an interior structuring of tumors could be observed. This was characterized by time dependent alterations of contrast enhancement with initial enhancement of the tumor rim followed by a centripetal progression of the signal. In one case of a strong central contrast enhancement (Figure 3B) the tumor was explanted, fixed and slices were analysed histologically after HE staining. A large central necrotic/fibrotic area could be observed surrounded by peripherally arranged vital tumor cells (Figure 3C).

### Monitoring of xenograft tumor growth

**Figure 4:**
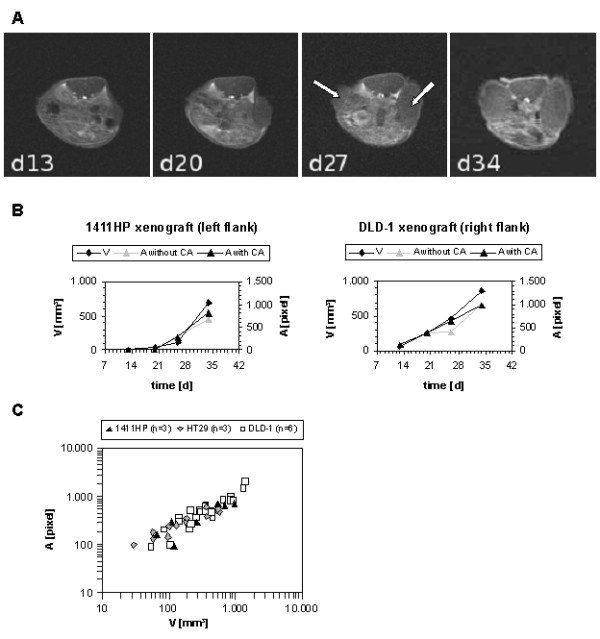
**Monitoring of xenograft tumor growth**. **(A): **Transaxial NMR images of a mouse (face-down position) bearing two s.c. xenografts (left: 1411HP germ cell tumor, right: DLD-1 colon carcinoma) analysed over 5 weeks (d13, d20, d27, d34 post cell injection). Depicted images were taken 10 min after i.v. application of Gd-BOPTA. White arrows point at tumors. **(B): **Following tumor growth of example shown in Figure 4A as analysed by calliper measurements and volume calculation compared to analyses by pixel extension of tumor sections based on NMR images (with or without Gd-BOPTA (CA)). Both tumor volume (V) and tumor section extent (A) comparably increased over the observation period. **(C): **Correlation of both methods: calculation of tumor growth by calliper measurement (V) and pixel extension analyses based on NMR images (A) of all 12 tumors.

Apart from tumor detection the quantification of tumor burden is one important aspect of non-invasive in vivo imaging techniques. To test whether the BT-MRI system is suitable for following s.c. xenograft growth the tumor burden was examined in 2 groups of 3 mice each bearing 2 different tumors: one group with 1411HP germ cell tumor and DLD-1 colon carcinoma, one group with HT29 colon carcinoma and DLD-1 colon carcinoma. Growth of tumors was followed using (a) calliper measurement and volume calculation and (b) BT-MRI and measurement of pixel extensions of tumor sections based on NMR images. For both methods comparable progression profiles could be observed, which was independent of Gd-BOPTA injection. A representative example of one individual is presented in Figure 4A and 4B. In addition, all values calculated by pixel extension analyses were plotted dependent on respective values calculated by calliper measurement. This demonstrates the correlation of both applications (Figure 4C).

## Discussion

MRI as a non-invasive imaging technology plays a key role in preclinical in vivo evaluation of tumor therapies. The development of a BT-MRI system for small animal imaging could lead to easy detection of tumor mass and progression with little effort and low costs. Additionally, MRI provides an insight into organs and tissues of laboratory animals.

The experimental results clearly proof that BT-MRI can be used to visualise organs and tumors in nude mouse xenograft models. Subcutaneous xenografts were easily identified as relative hypointense areas in transaxial slices of NMR images. In addition BT-MRI system is suitable for following xenograft tumor growth. Monitoring of tumor progression evaluated by pixel extension analyses based on NMR images correlated with increasing tumor volume calculated by calliper measurement. This is an important requirement for application of BT-MRI system in orthotopic/metastatic tumor models to evaluate the whole tumor burden. For this purpose it is necessary to take serial slices of NMR images to get the largest dimension of the tumor as basis for calculation. In addition the whole tumor shape can be reconstituted.

One critical aspect using orthotopic/metastatic tumor models could be the visualization of metastasis in tissues and organs depending on the model. This may require application of contrast agent for differentiation between tumor and normal tissue. In this study we used Gd-BOPTA as one of the clinically used low molecular weight gadolinium chelates. Gd chelates are commonly used as MRI contrast agents for the detection of solid tumors in patients where an initial tumor rim enhancement is usually observed [12-18]. Thereby the characteristic enhancement of the tumor rim can be used for the differentiation between malignant and benign masses [15]. Initially most tumors in our study showed no peripheral contrast enhancement on NMR images. Applying a higher but well tolerated dose of Gd-BOPTA such an effect could be observed, albeit not in each case. This may be due to the artificial location of the tumor as subcutaneous xenograft. Moreover, it was observed that low molar mass Gd chelates show an initial rim enhancement, followed by a washout effect, which requires that the images are obtained within the first 2 min after injection [19]. This probably explains the lack of initial rim enhancement in our models after application of low dose Gd-BOPTA. In this regard the application of macromolecular MRI contrast agents could be useful [20]. They have a longer circulation time and are more confined to the blood pool, therefore giving a longer time window for imaging in mice models.

A main advantage of MRI is the capability to characterize important tumor characteristics (e.g. internal structure, oedema in the tumor environment, necrotic areas). We observed a pronounced interior structuring of an s.c. HT29 tumor after i.v. injection of the contrast agent Gd-BOPTA. Histological analyses revealed that a large central necrotic/fibrotic area was associated with contrast enhancement. Such an effect can also be observed in patient tumors. After the characteristic initial tumor rim enhancement a centripetal progression of the signal can occur depending on the tumor structure, e.g. determined by different vascular architecture [12,15,21]. Early peripheral enhancement with centripetal progression was seen in invasive carcinomas with a high peripheral and a low central microvessel density, which was associated with fibrosis and/or necrosis [12,21]. This demonstrates that depending on the tumor and used contrast agent the BT-MRI system is suitable for observation of intratumoral structures and that characteristic features of patient tumors can be reproduced in the model system. It offers the opportunity to follow intratumoral processes under therapy.

Further work will be done particularly with regard to imaging of different orthotopic installed tumors and their progression as well as the development of metastatic disease. Other contrast agents will also be examined in order to find better enhancement of (small) tumor sites and metastases. Moreover, other contrast enhancer could lead to better results for imaging of interior tumor structures.

## Conclusions

The results of the current study show that BT-MRI is, despite its limitations with respect to the magnetic field strength and magnet homogeneity, clearly capable of providing satisfactory image slice quality to visualize organs and tumors and to monitor tumor progression in mouse models.

## List of abbreviations

MRI: magnetic resonance imaging; BT-MRI: benchtop-magnetic resonance imaging; NMR: nuclear magnetic resonance; Gd-BOPTA: gadobenate dimeglumine; s.c.: subcutaneous; HE: hematoxylin/eosin

## Competing interests

The authors declare that they have no competing interests.

## Authors' contributions

HC, HM, KM and TM designed the study. HC, HM and TM performed experiments. HC, HM, KM and TM analysed data. HC and TM wrote the paper. All gave final approval.
